# Specificity of Anti-Tau Antibodies when Analyzing Mice Models of Alzheimer's Disease: Problems and Solutions

**DOI:** 10.1371/journal.pone.0094251

**Published:** 2014-05-02

**Authors:** Franck R. Petry, Jérôme Pelletier, Alexis Bretteville, Françoise Morin, Frédéric Calon, Sébastien S. Hébert, Robert A. Whittington, Emmanuel Planel

**Affiliations:** 1 Université Laval, Faculté de Médecine, Départment de Psychiatrie et Neurosciences, Québec, Canada; 2 Université Laval, Faculté de Pharmacie, Québec, Canada; 3 Centre de Recherche du CHU de Québec, CHUL, Axe Neurosciences, Québec, Canada; 4 Department of Anesthesiology, College of Physicians and Surgeons, Columbia University, New York, New York, United States of America; CSIC/Universidad Autonoma Madrid, Spain

## Abstract

Aggregates of hyperphosphorylated tau protein are found in a group of diseases called tauopathies, which includes Alzheimer's disease. The causes and consequences of tau hyperphosphorylation are routinely investigated in laboratory animals. Mice are the models of choice as they are easily amenable to transgenic technology; consequently, their tau phosphorylation levels are frequently monitored by Western blotting using a panel of monoclonal/polyclonal anti-tau antibodies. Given that mouse secondary antibodies can recognize endogenous mouse immunoglobulins (Igs) and the possible lack of specificity with some polyclonal antibodies, non-specific signals are commonly observed. Here, we characterized the profiles of commonly used anti-tau antibodies in four different mouse models: non-transgenic mice, tau knock-out (TKO) mice, 3xTg-AD mice, and hypothermic mice, the latter a positive control for tau hyperphosphorylation. We identified 3 tau monoclonal antibody categories: type 1, characterized by high non-specificity (AT8, AT180, MC1, MC6, TG-3), type 2, demonstrating low non-specificity (AT270, CP13, CP27, Tau12, TG5), and type 3, with no non-specific signal (DA9, PHF-1, Tau1, Tau46). For polyclonal anti-tau antibodies, some displayed non-specificity (pS262, pS409) while others did not (pS199, pT205, pS396, pS404, pS422, A0024). With monoclonal antibodies, most of the interfering signal was due to endogenous Igs and could be eliminated by different techniques: i) using secondary antibodies designed to bind only non-denatured Igs, ii) preparation of a heat-stable fraction, iii) clearing Igs from the homogenates, and iv) using secondary antibodies that only bind the light chain of Igs. All of these techniques removed the non-specific signal; however, the first and the last methods were easier and more reliable. Overall, our study demonstrates a high risk of artefactual signal when performing Western blotting with routinely used anti-tau antibodies, and proposes several solutions to avoid non-specific results. We strongly recommend the use of negative (i.e., TKO) and positive (i.e., hypothermic) controls in all experiments.

## Introduction

Alzheimer's disease (AD) is the most common neurodegenerative disease and is characterized by a progressive loss of cognitive function, leading to dementia [1], [2]. The two neuropathological hallmarks of AD are extracellular senile plaques composed of aggregates of amyloid-beta protein (Aβ; [2]) and intracellular neurofibrillary tangles (NFTs) composed of aggregates of the hyperphosphorylated microtubule-associated protein tau [3], [4]. Tau plays a role in promoting the assembly and maintenance of microtubules through its microtubule-binding domain. The capacity of tau to bind microtubules and promote stabilization and assembly is negatively regulated by its phosphorylation, particularly in and around the microtubule binding domain [5]. Under pathological conditions, such as AD and others tauopathies, tau becomes hyperphosphorylated, resulting in reduced affinity for microtubules and self-aggregation into abnormal filaments, leading to formation of NFTs [6]. The etiology and consequences of tau hyperphosphorylation are not well understood and are routinely investigated in laboratory animals. Mice are the models of choice for this kind of research, as they are easily amenable to the application of transgenic technologies. Thus, the analysis of tau phosphorylation levels by Western blotting is commonly used to assess tau pathology and to better understand the link between phosphorylation and the events that occur in tauopathies. Nevertheless, the use of antibodies can lead to non-specific results. Indeed, in mouse studies, secondary anti-mouse antibodies can bind to endogenous immunoglobulins (Igs), which are present within brain tissues [7], thus interfering with the tau signal produced by mouse monoclonal primary antibodies. Moreover, primary anti-tau rabbit polyclonal antibodies can recognize other proteins, with similar molecular weights to that of tau, leading to non-specific bands masking or interfering with the tau signal.

The purpose of this study was to identify the specific and non-specific signals for a panel of commonly used anti-tau antibodies. Hence, we compared the anti-tau antibody immunoreactivity profile in 4 mouse models: non-transgenic wild-type mice (WT) expressing endogenous murine tau with low levels of tau phosphorylation, tau knock-out (TKO; [8]) mice invalidated for their murine tau gene as a negative control for the detection of non-specific signal, 3xTg-AD mice [9] that express human mutated tau protein (P301L) as well as human mutated amyloid precursor protein (APPswe) on human mutated presenilin 1 (PS1) background. Finally, anesthetized C57BL/6J mice were used as a positive control, as we have previously shown that anesthesia-induced hypothermia induces tau hyperphosphorylation (phospho-tau [10]).

Our results revealed different tau phosphorylation signal (band) profiles in the 4 mouse models when monoclonal antibodies were used. The use of secondary antibodies specific to native Igs or the light chain of Igs completely removed the non-specific signal, while techniques used for removing Igs also abolished non-specific signals. Finally, some polyclonal antibodies produced several non-specific bands that masked the tau signal.

## Materials and Methods

### Animals and treatment

Animals were handled according to procedures approved by the Comité de Protection des Animaux du CHUL under the guidelines of the Canadian Council on Animal Care. Mice were maintained in a temperature-controlled room (∼23°C) with a light/dark cycle of 12/12 h and had access to food and water *ad libitum*. We used 4 groups of mice: TKO, 3xTg-AD, WT and hypothermic mice. TKO mice (*Mapt^tm1(EGFP)Klt^*/J, # 004779, Jackson Laboratory, Bar Harbor, ME, USA) have a knock-in of the EGFP coding sequence into the first exon that disrupts the expression of the *Mapt* gene and produces a cytoplasmic EGFP fused to the first 31 amino acids of tau protein. We used them in this study as negative controls to visualize non-specific signals due to their absence of tau protein [8], [11]. As positive control for human tau, we used 3xTg-AD mice (generous gift of Dr. Frank LaFerla, University of California, Irvine, USA), which express 2 major mutations causally linked to familial AD APP_Swe_, PS1_M146V_, as well as tau_P301L_ which is the proline to leucine mutation at the 301 codon of tau that has been causally associated with frontotemporal dementia and parkinsonism linked to chromosome 17 (FTDP-17) [9]. As the 3xTg-AD mice are on a C57BL/6;129X1/SvJ;129S1/Sv genetic background, we used wild-type mice of the same background as non-transgenic controls (WT). As a positive control for tau hyperphosphorylation, we intra-peritoneally injected C57BL/6J mice (# 000664, Jackson Laboratory) with a mixture of xylazine at 10 mg/kg and ketamine at 100 mg/kg for 30 min, without temperature control, to induce hypothermia-mediated tau hyperphosphorylation [10]. In this study all animals were 18 month-old male mice except for the hypothermic group, which consisted of 2 month-old males (n = 3 for each group). All mice were killed by decapitation without anesthesia to avoid transient changes in tau phosphorylation [10] and the brains were rapidly dissected on ice and then frozen on dry ice.

### Immunoblot analysis

Frozen cortices were processed as described [12]. Briefly, they were homogenized in cold radioimmunoassay precipitation (RIPA) buffer (50 mM Trish-HCl, pH 7,4, 1% NP-40, 150 mM NaCl, 0,25% Na-Deoxycholate, 1 mM EDTA, 1 mM Na3VO4, 1 mM NaF, 1 mM PMSF and 10 µl/ml of proteases inhibitors P8340, Sigma Aldrich, Saint-Louis, MO, USA) with a mechanical homogenizer and then centrifuged at 20,000 g at 4°C for 20 min to isolate the soluble proteins. The proteins were mixed with a Western blot loading sample buffer (NuPAGE LDS; Invitrogen, Carlsbad, CA, USA) containing 5% of 2-mercapto-ethanol and boiled for 10 minutes. For each sample, proteins were separated by sodium dodecyl sulfate polyacrylamide gel electrophoresis (SDS-PAGE) using Tris-Glycine gels containing 10% acrylamide/bis-acrylamide and then blotted onto nitrocellulose membranes (Hybond, GE Healthcare, Freiburg, Germany). The amount of protein loaded is indicated in Table 1. Precision Plus Protein Standards (#161-0373; Biorad, Hercules, CA, USA) were used as protein molecular weight indicators. After blocking with 5% dry milk for 1 h at room temperature, membranes were incubated overnight at 4°C with primary antibodies diluted in Superblock blocking buffer (Thermo Fisher Scientific, Rockford, IL, USA) at the concentration indicated in Table 1. The following day, the membranes were washed 3 times with phosphate buffered saline solution with 0.1% tween and then incubated with a horseradish peroxidase-coupled secondary antibody (Goat anti-mouse #115-035-003 and Goat anti-rabbit #111-035-003, Jackson Immunoresearch Laboratories, West Grove, PA, USA) or Mouse TrueBlot ULTRA antibody (anti-mouse Ig HRP 18-8817-33, eBiosciences, San Diego, USA). For the heat stable fraction, conventional secondary antibodies were used. Finally, peroxidase activity was revealed using an ECL (Enhanced Chemiluminescence) detection kit (Millipore, Billerica, MA, USA) and visualized with a Fujifilm LAS4000 imaging system (Fujifilm Life Science USA, Stamford, CT).

**Table 1:** Antibodies used in this study.

Antibody	Epitope	Supplier (catalog#)	Lot number	Dilution	Non-specificity	Proteins loaded (µg)/Incubation primary/Incubation secondary			
					displayed	HC+LC Ab	TB Ab	HS fraction	LC-specific Ab
**Mouse monoclonal primary antibodies**									
**AT8**	pSer202-pThr205	Fisher (MN1020)	ND 169248	1/1000	high	5/ON/1h	15/2d/3d	10/ON/1h	20/ON/ON
**AT180**	pThr231	Fisher (MN1040)	JK 128858	1/1000	high	5/ON/1h	5/ON/1h	10/ON/1h	-
**MC1**	Tau 5-15/312-322	Peter Davies	-	1/1000	high	5/ON/1h	5/ON/1h	15/ON/1h	-
**MC6**	pSer235	Peter Davies	-	1/1000	high	5/ON/1h	5/ON/1h	15/ON/1h	-
**TG-3**	pThr231	Peter Davies	-	1/1000	high	5/ON/1h	5/ON/1h	10/ON/1h	-
**AT270**	pThr181	Fisher (MN1050)	JK 128859	1/1000	moderate	15/ON/1h	15/ON/ON	10/ON/1h	20/ON/ON
**Tau12**	Tau 9-18 (human)	Abcam (74137)	GR 26877-7	1/1000	moderate	5/ON/1h	5/ON/1h	5/ON/1h	-
**TG-5**	Tau 220-242	Peter Davies	-	1/1000	moderate	5/ON/1h	5/ON/1h	5/ON/1h	-
**CP13**	pSer202	Peter Davies	-	1/1000	moderate	5/ON/1h	5/ON/1h	10/ON/1h	-
**CP27**	Tau 130-150 (human)	Peter Davies	-	1/1000	moderate	5/ON/1h	5/ON/1h	10/ON/1h	-
**Tau-1**	depSer195/198/199/202	Millipore (MAB3420)	JK 1958299	1/1000	absent	5/ON/1h	5/ON/1h	5/ON/1h	-
**Tau46**	Tau 404-441	Abcam (22261)	GR 54105-1	1/1000	absent	5/ON/1h	5/ON/1h	5/ON/1h	20/ON/ON
**PHF-1**	pSer396/pSer404	Peter Davies	-	1/1000	absent	5/ON/1h	5/ON/1h	5/ON/1h	-
**DA9**	Tau 102-140	Peter Davies	-	1/1000	absent	5/ON/1h	5/ON/1h	10/ON/1h	-
**Rabbit polyclonal primary antibodies**									
**Total Tau**	Tau 243-441	Dako (A0024)	61284	1/2000	-	5/ON/1h	-	-	-
**pS199**	Ser199	Invitrogen (44734G)	786604C	1/2000	-	5/ON/1h	-	-	-
**pS262**	Ser262	Invitrogen (44750G)	846797B	1/2000	high	5/ON/1h	-	20/ON/1h	-
**pS396**	Ser396	Invitrogen (44752G)	567847A	1/2000	-	5/ON/1h	-	-	-
**pS404**	Ser404	Invitrogen (44758G)	0400E	1/1000	-	5/ON/1h	-	-	-
**pS409**	Ser409	Invitrogen (44760G)	0500D	1/1000	high	5/ON/1h	-	20/ON/1h	-
**pS422**	Ser422	Invitrogen (44774G)	883416A	1/1000	-	5/ON/1h	-	-	-
**pT205**	Thr205	Invitrogen (44738G)	519317B	1/2000	-	5/ON/1h	-	-	-
**Secondary antibodies**									
**HC+LC**	goat anti-rabbit Ig HRP	Jackson laboratory	102587	1/5000	-	1/5000	-	1/5000	-
**HC+LC**	goat anti-mouse Ig HRP	Jackson laboratory	97103	1/5000	-	1/5000	-	1/5000	-
**TrueBlot**	anti-mouse Ig HRP	eBiosciences (18-8817-33)	E07430-1635	1/1000	-	-	1/1000	-	-
**LC-specific Ab**	goat anti-mouse Ig HRP	Millipore (AP200P)	2297170	1/5000	-	-	-	-	1/5000

Information about all used antibodies in this study is reported including the epitope recognized, the antibody supplier, the catalog number, the lot number and the dilution used in Western Blot procedure. Antibodies were divided in 3 categories: primary monoclonal antibodies, primary polyclonal antibodies and secondary antibodies. Membranes were incubated overnight (ON) or over 2 days (2d) with primary antibodies at 4°C. The following day, membranes were washed 3 times and then incubated with a horseradish peroxidase-coupled secondary anti-mouse/rabbit antibody that recognizes both heavy chain (HC) and light chain (LC) of Igs, or TrueBlot ULTRA antibody, or light chain (LC)-specific antibody. Information regarding the amount of homogenate proteins loaded per lane for each antibody, as well as the incubation time for primary and secondary antibodies is also reported. We classified monoclonal antibodies according to their non-specificity against endogenous Igs; high non-specificity (black), moderate (grey), and absent (white). Antibodies in white can be detected with conventional secondary anti-mouse antibodies (HC+LC) because they do not display non-specificity. Antibodies in grey display low non-specificity and secondary anti-mouse antibodies can be used (HC+LC). Eventually, antibodies in black display high non-specificity and TrueBlot or anti-LC secondary antibodies are necessary to detect tau signal.

### Heat stable fraction

After homogenization in cold RIPA buffer and centrifugation, 20 µl of the supernatant containing the proteins was boiled at 95°C for 10 min and then centrifuged at 20,000 g at 4°C for 10 min. The supernatant corresponding to the heat stable (HS) fraction was then harvested. This method is used to isolate proteins resistant to heat including tau and other microtubule-associated proteins (MAPs) [13]. Thus, endogenous Igs were precipitated during the boiling process and eliminated from the supernatant. The proteins were then mixed with Western blotting sample loading buffer (NuPAGE LDS, Invitrogen) containing 5% 2-mercaptoethanol and boiled for 10 min. To determine whether the HS fraction induced tau protein loss during the boiling and centrifugation steps, we used 3 brain samples from control mice (WT) and boiled the supernatant containing the proteins for 5 min, to obtain an HS fraction. Then the samples were centrifuged at 20,000 g at 4°C for 10 min, the supernatant harvested, and the proteins were mixed with Western blotting sample loading buffer (NuPAGE LDS, Invitrogen) containing 5% 2-mercaptoethanol and boiled for 10 min, resulting in the HS fraction. The total supernatant after homogenization was mixed with Western blotting sample loading buffer (NuPAGE LDS, Invitrogen) containing 5% 2-mercaptoethanol and boiled for 10 min, resulting in the total fraction. Finally, the pellet was mixed with Western blotting sample loading buffer (NuPAGE LDS, Invitrogen) containing 5% 2-mercaptoethanol, homogenized and then boiled for 10 min, constituting the pellet fraction. We compared these 3 fractions, total, HS, and pellet by Western blotting and incubated them with total tau, ERK and GAPDH antibodies.

### Protein G

After homogenization in cold RIPA buffer and centrifugation, 25 µl of the supernatant containing the proteins were mixed with 5 µl of protein G-Plus agarose suspension (Cat# IP04, EMD Chemicals, Inc. San Diego, CA) and incubated at 4°C for 1h. The mixture was then centrifuged at 20,000g at 4°C for 20 min to precipitate protein G so as to clear the supernatant of endogenous Igs. The proteins were then mixed with sample loading buffer (NuPAGE LDS, Invitrogen) containing 5% 2-mercaptoethanol and boiled for 10 min.

### Dynabeads

After homogenization in cold RIPA buffer and centrifugation, 20 µl of the supernatant containing the proteins were mixed with 5 µl of Dynabeads M-280 Sheep anti-mouse IgG (Cat# 112.01D, Invitrogen Dynal AS, Oslo, Norway) and incubated at 4°C for 1 h. The mixture was then suspended in a magnetic particle concentrator (DynaMag-2, Cat# 123.21D, Invitrogen Dynal AS) to separate the beads from the solution. The supernatant was harvested and, at this point, it was cleared of endogenous Igs. The proteins were then mixed with sample loading buffer (NuPAGE LDS, Invitrogen) containing 5% 2-mercaptoethanol and boiled for 10 minutes.

### Antibodies

**Figure 1:**
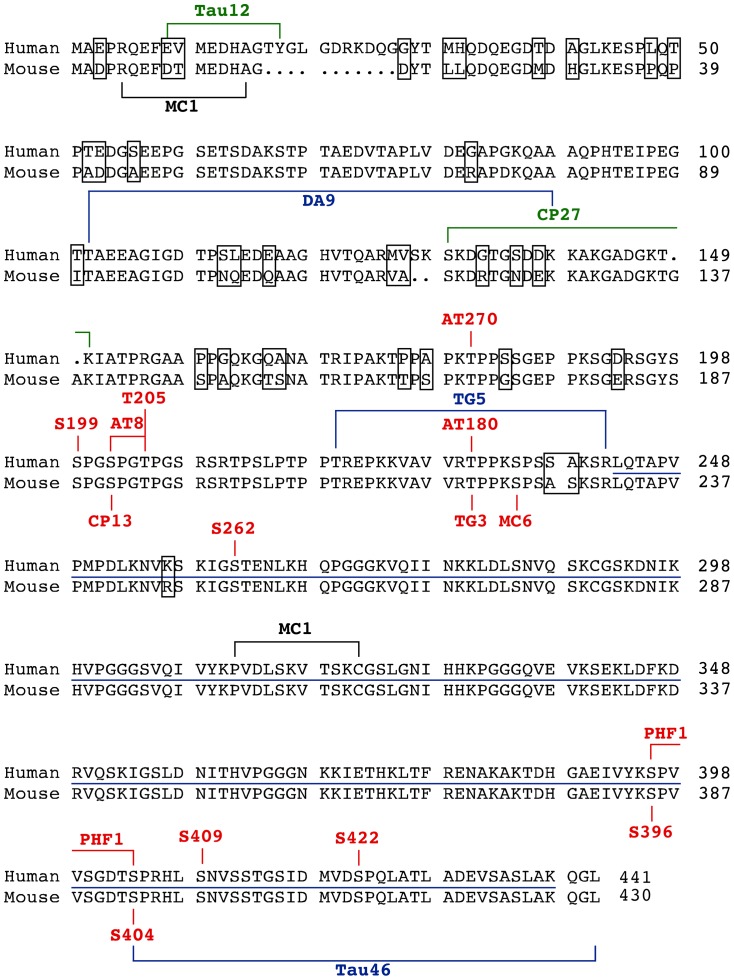
Comparison between human and mouse tau amino acidic sequence. We reported the full-length amino acid sequence of the human and mouse tau protein containing the alternative spliced exon 2,3 and 10. We also indicated the epitopes recognized by each anti-tau primary antibodies used in this study: red: phosphorylated epitopes, black: conformational epitope, blue: human and mouse tau epitopes, green: human tau epitopes. The line between the human and mouse sequences corresponds to the sequence of amino acid recognized by the total tau primary antibody. Points correspond to a missing sequence in the species and black frames correspond to amino acid sequence differences between human and mouse species.

The following commercial anti-tau monoclonal antibodies were used: AT8 (pSer^202^/pThr^202^ Fisher cat# MN1020), AT100 (pSer^212^/pSer^214^ Fisher cat# MN1060), AT180 (pThr^231^ Fisher cat# MN1040), AT270 (pThr^181^ Fisher cat# MN1050), Tau-1 (de-phosphorylated Ser^195^/Ser^198^/Ser^199^/Ser^202^ Millipore cat# MAB3420), Tau12 (human Tau^9-18^ Abcam cat# 74137), and Tau46 (Tau^404-441^ Abcam cat# 22261). The non-commercial monoclonal antibodies were a generous gift of Dr. Peter Davies (Feinstein Institute for Medical Research, Manhasset, NY, USA): CP13 (pSer^202^), CP27 (human Tau^130-150^), DA9 (Tau^102-140^), MC1 (Tau^5-15/312-322^ conformational antibody), MC6 (pSer^235^), PHF-1 (pSer^396^/pSer^404^), TG3 (pThr^231^) and TG5 (Tau ^220-242^) [14], [15], [16], [17], [18], [19], [20], [21]. Purified rabbit polyclonal phospho-tau antibodies were purchased from Invitrogen: anti-pSer^199^ (44734G), pSer^262^ (44750G), pSer^396^ (44752G), pSer^404^ (44758G), pSer^409^ (44760G), pSer^422^ (44774G) and pThr^205^ (44738G). The total tau antibody was purchased from Dako (hTau^243-441^, #cat A0024). GAPDH antibody was purchased from Millipore (#cat MAB374, clone 6C5). HRP- coupled anti-mouse and anti-rabbit secondary antibodies were purchased from Jackson Laboratory. HRP-coupled anti-mouse TrueBlot secondary antibody, which consists of specific secondary antibodies that only recognize the native form of Igs was purchased from eBiosciences (#cat 18-8817-33). HRP-coupled goat anti-mouse Light Chain (LC) antibody was from Millipore (#cat AP200P). This antibody reacts strongly with native primary antibodies primarily with kappa light chains and does not detect heavy chains. A summary of all the antibodies and dilutions used is available in Table 1, and a map of all the epitopes is displayed in Figure 1.

### Statistical Analysis

Statistical analysis was performed with 1-way ANOVA followed by a Newman-Keuls Multiple Comparison Test. Data are expressed as mean ± SD. * denotes a significant difference compared to WT with P<0,05, ** with P<0,01 and *** with P<0,001. # denotes a significant difference compared to 3xTg-AD with P<0,05, ## with P<0,01 and ### with P<0,001. n = 3 for each condition. For figures without quantifications, quantification data are available as Supplementary Figures.

## Results

### Tau signal analysis with commercial monoclonal antibodies

**Figure 2:**
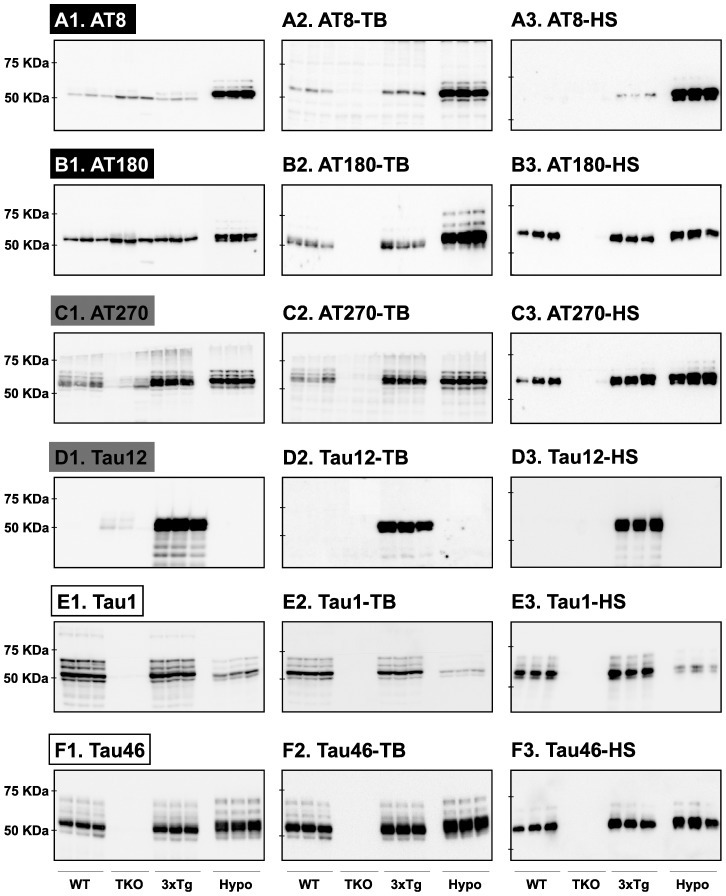
Analysis of tau signal with commercial monoclonal antibodies by Western blotting. The proteins were extracted form the cortex of 3 mouse lines: control mice (WT and Hypothermic), Tau KO mice and 3xTg mice with n = 3 for each group. Proteins were extracted with SDS-PAGE and then identified with the following commercial monoclonal antibodies: A: AT8, B: AT180, C: AT270, D: Tau 12, E: Tau1 and F: Tau 46. Normal anti-mouse (1) and Mouse TrueBlot ULTRA (2) secondary antibodies were used to detect primary antibodies. The heat stable fraction was used to remove non-specificity (3). Quantifications of the blots are available in Figure S1.

We first aimed to determine the specificity of commercial anti-tau monoclonal antibodies (Table 1). A representative Western blot analysis of tau protein in cortical tissue is shown in Figure 2 (A1–F1). Quantification of the blots is available in Figure S1. Based on these results, it was possible to classify the anti-tau antibodies according to 3 distinct immunoblot staining profiles. In the first category of antibodies (i.e., labeled in black), which was observed using the AT8 and AT180 epitopes, non-specific bands were detected at ∼50 kDa in TKO mice (Fig. 2A1, B1). In the anesthetized hypothermic mice, a different pattern was observed. Here, a strong signal was observed at ∼50 kDa with additional, weaker bands at ∼55–60 kDa (Fig. 2A1, B1) that, together, correspond to the 3 tau isoforms of murine tau. Thus, under basal conditions the non-specificity of AT8 and AT180 anti-tau antibodies is high.

In the second antibody category (i.e., labeled in grey), which was observed using the AT270 and Tau12 epitopes, weak non-specific bands were observed at ∼50 kDa in TKO mice (Fig. 2C1, D1). It should be noticed that Tau12 recognizes specifically human Tau, consistent with the literature. Interestingly, no considerable increase in AT270 signal was observed in the hypothermic when compared to 3xTg-AD mice (a common feature of several anti-tau phospho-antibodies, see below). Taken together, these observations demonstrate that both AT270 and Tau12 anti-tau antibodies produce, to some degree, non-specific signals.

In the third antibody category (i.e., labeled in white), which was observed using the Tau1 and Tau46 epitopes, no signal was observed at all in the TKO mice (Fig. 2E1, F1), thus indicating the high specificity of these antibodies under basal conditions. Overall, these results show the various degrees of specificity of commonly used anti-Tau antibodies.

We next investigated whether avoiding signal from endogenous immunoglobulins (Igs) would improve the specificity of anti-tau monoclonal antibodies. To accomplish this, we used TrueBlot secondary antibody (see methods). With the first category of antibodies, use of the TrueBlot secondary antibody completely removed non-specific signals (Fig. 2A2, B2). Notably, the non-specific signals were also removed in both WT and 3xTg-AD mice (Fig. 2A2, B2) allowing for the restoration of the “true” tau signal. Finally, there was no change of tau phosphorylation levels in hypothermic mice with TrueBlot treatment. With the second category of antibodies, TrueBlot treatment produced similar effects in terms of removing the non-specific signals. Indeed, the non-specific bands observed in the TKO mice were completely abolished (Fig. 2C2, D2). As expected, similar results were obtained with the third antibody category (Fig. 2E2, F2). Therefore, the TrueBlot secondary antibody restored specific tau signals.

To further confirm that endogenous Igs produced the non-specific anti-tau signals, we tested the same antibodies in heat-stable protein fractions (see methods). Since native tau protein forms a linear structure in solution, it is less prone to aggregation at high temperature. This is in sharp contrast to Igs, which harbor complex tertiary structures, and thus, precipitate following exposure to heat (i.e., boiling). Consistent with our hypothesis, no signal was observed in TKO mice, regardless of the antibodies and degree of non-specificity observed previously (Fig. 2A3-F3). It's noteworthy that some phospho-tau signals were less intense following heat treatment (Fig. 2A3, B3, E3 and F3), suggesting that this approach might induce a certain loss of tau. Nevertheless, in addition to validating our hypothesis, we provide a cost-effective and simple alternative to TrueBlot antibodies for specifically measuring tau phosphorylation in mice using commercial monoclonal antibodies.

### Tau signal analysis with non-commercial monoclonal antibodies

**Figure 3:**
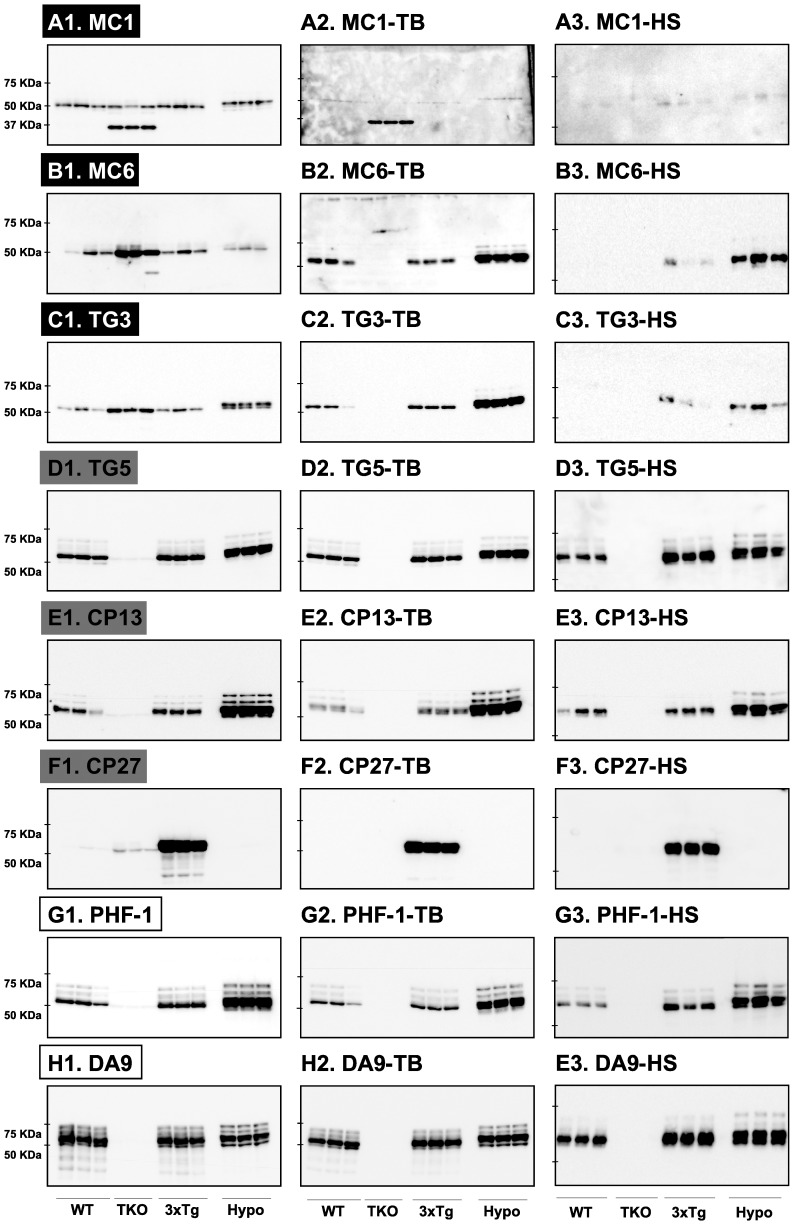
Analysis of tau signal with non-commercial monoclonal antibodies by Western blotting. The proteins were extracted form the cortex of 3 mouse lines: control mice (WT and Hypothermic), Tau KO mice and 3xTg mice with n = 3 for each group. Proteins were extracted with SDS-PAGE and then identified with the following commercial monoclonal antibodies: A: MC1, B: MC6, C: TG3, D: TG5, E: CP13, F: CP27, G: PHF-1, H: DA9. Normal anti-mouse (1) and Mouse TrueBlot ULTRA (2) secondary antibodies were used to detect primary antibodies. The heat stable fraction was used to remove non-specificity (3). Quantifications of the blots are available in Figure S2.

**Figure 4:**
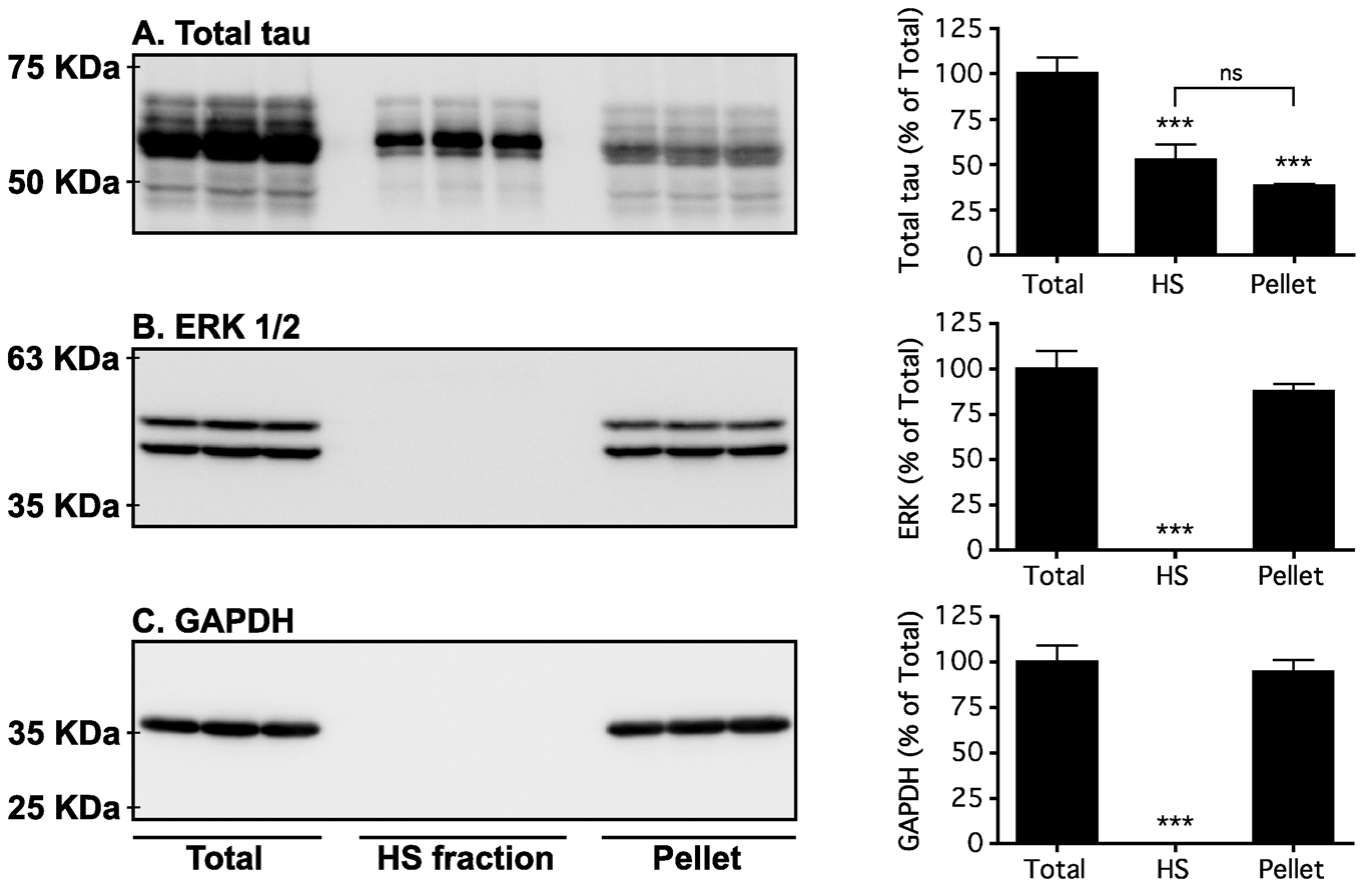
Effect of HS procedure on protein levels. To determine the effect of HS procedure on protein levels, we compared the signal of Total tau (A), ERK 1/2 (B) and GAPDH (C) in control mice (WT) under 3 conditions: total sample (Total), HS fraction, and the pellet obtain after centrifugation. Data are expressed as mean ± SD, *** denotes a significant difference versus Total with P<0,001, n = 3 for each condition.

We next performed similar experiments using non-commercial anti-tau monoclonal antibodies (Table 1). As expected, these results were comparable with those obtained using commercial anti-tau monoclonal antibodies. The first antibody category included MC1, MC6 and TG3 epitopes (Fig. 3A1, B1, 2C1). With MC6 and TG3 anti-tau antibodies, the signal was mainly evident in hypothermic mice (Fig. 3A1, C1, D1). The second antibody category included TG5, CP13, and CP27 epitopes (Fig. 3D1, E1, F1). Finally, the third category of antibodies included PHF-1 and DA9 epitopes (Fig. 3G1, H1). As before, TrueBlot treatment removed non-specific signals due to endogenous Igs (Fig. 3A2–C2). Quantification of the blots is available in Figure S2. The HS fraction could also remove the non-specific signals to various degrees (Fig. 3A3–F3). Here again, we observed a less intense tau signal. To investigate whether this was due to a loss of tau during the HS preparation, we blotted the total, HS, as well as the pellet fractions and examined the total tau signal. We found that about 50% of total tau signal was lost in the pellet (Fig. 4A). To verify whether the HS procedure was done correctly, we also blotted for ERK and GAPDH, two proteins that should have precipitated during the procedure. Indeed, the total amount of the two proteins was collected in the pellet and no signal was detected in the HS fraction (Fig. 4B, C). Thus, although HS preparation is a good way to investigate a fraction of tau that is very soluble and heat-resistant, it also leads to a loss of tau in the pellet. This probably occurs because tau gets trapped with other proteins during the denaturation process. Overall, similar results were observed using both commercial and non-commercial mouse monoclonal anti-tau antibodies.

### Tau antibody specificity following clearance of Igs

**Figure 5:**
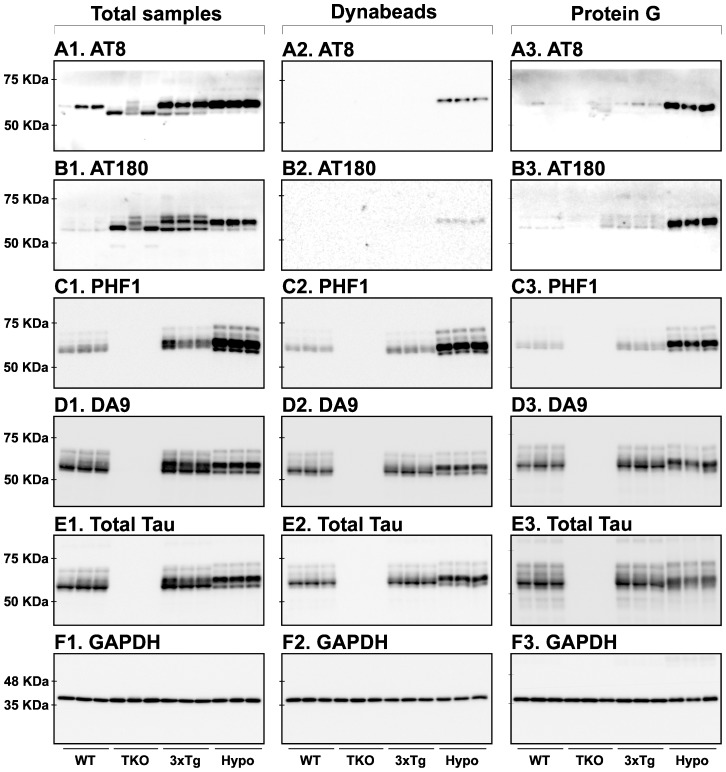
Effect of the preclearing on tau signal by Western blotting. The proteins were extracted form the cortex of 3 mouse lines: control mice (WT and Hypothermic), Tau KO mice and 3xTg mice with n = 3 for each group. The supernatant obtained from tissue homogenization was processed either in the normal manner to obtain total (non-cleared) sample (1) or processed to prepare cleared samples with Dynabeads (2) or protein G (3). Protein were extracted with SDS-PAGE and then identified with the following antibodies for both total and cleared samples: A: AT8, B: AT180, C: PHF-1, D: DA9, E: total tau, F: GAPDH. Quantifications of the blots are available in Figure S3.

To demonstrate that the non-specific signal at 50 kD was due to endogenous Igs, we used Dynabeads or conventional Protein G agarose to bind to (and clear) endogenous Igs (see methods). Following a pre-incubation (pre-clear) with these products on the supernatants, Western blot analysis was performed using anti-tau antibodies. These results were compared to total, non-cleared supernatants. We selected AT8 and AT180 epitopes as these gave the highest non-specific signals (Fig. 2). Total tau and DA9 were used as internal controls to determine whether Dynabeads and Protein G altered total tau levels. GAPDH antibodies were used as loading control and revealed that the same amount of protein was loaded for each sample, as the signal of GAPDH did not change (Fig. 5F1–F3).

We first observed that the use of Dynabeads did not substantially modify the total tau signals in all mouse models tested (Fig. 5D2, E2 compared to 5D1, E1). Quantification of the blots is available in Figure S3. Interestingly, when Dynabeads-cleared supernatants were incubated with AT8, the tau phosphorylation signal appeared only in hypothermic mice (Fig. 5A2). These results strongly suggest that the bands observed in WT and 3xTg-AD mice correspond to endogenous Igs, at least under these conditions. Similar tau bands were observed using the AT180 antibody (Fig. 5B1). It should be noted that some tau-specific signals were observed using PHF1 and DA9 antibodies. Finally, we performed these experiments using Protein G, which gave overall similar results (Fig. 5A3–E3). Together, these experiments demonstrate that pre-incubation of anti-tau antibodies with either Dynabeads or Protein G agarose greatly reduces or removes non-specific signals produced by endogenous mouse Igs.

### Antibodies directed against light chain of Igs also removed the non-specific signals

**Figure 6:**
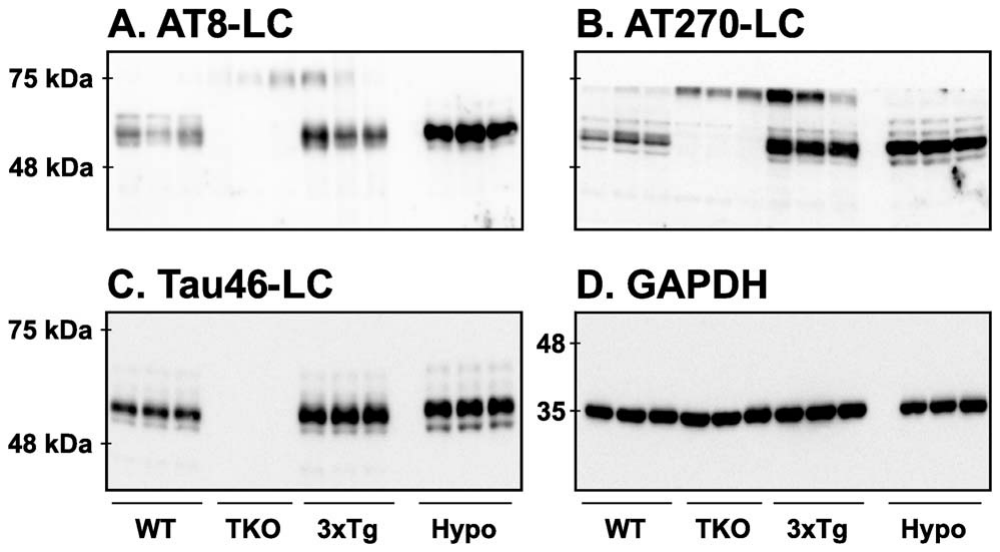
Analysis of tau signal with anti-mouse light chain secondary antibodies by Western blotting. The proteins were extracted form the cortex of 3 mouse lines: control mice (WT and Hypothermic), Tau KO mice and 3xTg mice with n = 3 for each group. Proteins were extracted with SDS-PAGE and then identified with the following commercial monoclonal antibodies: A: AT8, B: AT270, C: Tau46 and D: GAPDH. We used one antibodies of each non-specificity category (AT8 for high non-specificity, AT270 for moderate non-specificity and Tau46 for absence of non-specificity). Anti-mouse light chain secondary antibodies were used to detect primary antibodies except for GAPDH. Quantifications of the blots are available in Figure S4.

We used another kind of secondary antibody to remove the non-specificity coming from the heavy chain of mouse Igs. Indeed, some secondary antibodies were designed to react strongly with the kappa light chain of Igs but not with the heavy chain of Igs. We tested light chain antibodies (LC) with one commercial monoclonal primary antibody of each category: AT8 for the first category, AT270 for the second category and Tau46 for the third category (Fig 6). Quantification of the blots is available in Figure S4. With the first category, LC antibodies completely removed the non-specific signals (Fig. 6A). Notably, the non-specific signals were also removed in both WT and 3xTg-AD mice (Fig. 6A) allowing for the restoration of the “true” tau signal. With the second category of antibodies, LC antibodies produced similar effects in terms of removing the non-specific signals. Indeed, the non-specific bands observed in the TKO mice were completely abolished (Fig. 6B). As expected, similar results were obtained with the third antibody category (Fig. 6C). Therefore, using anti-LC secondary antibody restored specific tau signals.

### Tau signal analysis with polyclonal antibodies

**Figure 7:**
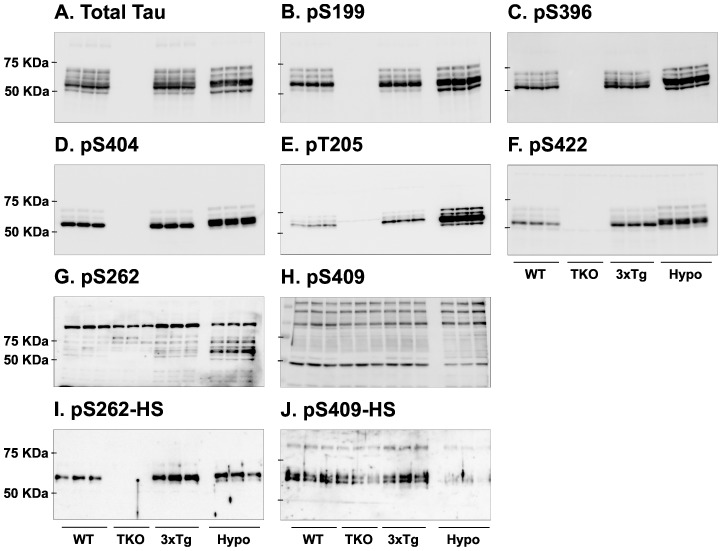
Analysis of tau signal with polyclonal antibodies by Western blotting. Proteins were extracted from the cortex of 3 mouse lines: control mice (WT and Hypothermic), Tau KO mice and 3xTg-AD mice. Proteins were separated by SDS-PAGE and then identified with the following polyclonal antibodies: A: Total Tau, B: pS199, C: pS396, D: pS404, E: pT205, F: pS422, G: pS262 and H: pS409. Normal anti-rabbit secondary antibodies were used to detect primary antibodies. The heat stable fraction was used to remove non-specificity: I: pS262 and J: pS409. Quantifications of the blots are available in Figure S5.

We finally performed Western blot analysis to examine the specificity of polyclonal anti-tau antibodies (Table 1). With almost all polyclonal antibodies, the non-specific bands observed previously in TKO mice did not appear (Fig. 7A, B, C). Quantification of the blots is available in Figure S5. Moreover, for most antibodies tested, the tau signal was clearly apparent above 50 kDa in WT and 3xTg-AD mice without interference from others proteins. However, some epitopes such as pS262 and pS409 produced non-specific bands at different molecular weights (Fig. 7G, H). In an attempt to improve the immuno-reactivity of these latter antibodies, we tested them in HS fraction [16]. Here, heat treatment greatly improved pS262, but not pS409, specificity (Fig. 7I, J).

## Discussion

In this study, we have demonstrated that several monoclonal antibodies directed against tau or phospho-tau epitopes can display non-specificity due to the property of secondary anti-mouse antibodies to bind to endogenous mouse Igs. When non-specificity was observed with monoclonal antibodies, we showed that several biochemical solutions could be used to remove the non-specific signal and improve the true tau signal.

The Western blot technique uses a SDS-PAGE to separate proteins, which are subsequently transferred on nitrocellulose membrane and identified with specific antibodies [22], [23], [24]. Because of the inclusion of blood-borne molecules, total brain lysates from mouse contain both tau protein and mouse Igs which display similar molecular weight: while the light chains of Igs have an apparent molecular weight around 25 kD and do not interfere with tau signal, the heavy chains migrate around 50 kD [25], which is where the majority of mouse tau isoforms are found [26]. One solution would be to remove the blood from the animals. However, this procedure requires anesthesia that we wanted to avoid because this procedure promotes tau hyperphosphorylation [10]. In addition, even after intracardiac perfusion, significant concentrations of mouse IgG (>10 ng/mg of wet tissue) can be found in cerebral homogenates [7]. Here, the problem of detection comes from the fact that the secondary anti-mouse antibody (designed to bind the heavy chain and the light chain of mouse immunoglobulins) recognizes both the primary antibody bound to tau and the endogenous Igs.

Using tau KO mice as a negative control, we found that the mouse primary antibodies could be classified into 3 categories according to the level (intensity) of non-specific signal observed with the tau KO samples: i) a high level of non-specificity, ii) moderate non-specificity, and iii) the absence of non-specificity. These non-specific signals are mostly observed with antibodies directed to phosphorylation sites. Thus, the intensity of the non-specific signal at 50 kD is not due to the difference in intrinsic specificity for tau epitopes between the primary antibodies, but rather due to the difference of in the abundance of a particular epitope in adult mouse brain. Indeed, some studies have compared the levels of phosphorylation of human tau between newborn brain, adult brain and AD brain and found that less of 5% of tau was phosphorylated in adult brain compared to 20% and 100% for newborn and AD brain respectively, indicating that adult tau protein is not extensively phosphorylated [27]. Furthermore, developmental studies in rodents have shown that while some epitopes, such as pT205 or pT181, are highly phosphorylated in the post-embryonic brain, their signal is very faint in adult brain [28], which could explain why AT8 (pS202/pT205) and AT270 (pT181) are more prone to display non-specific Igs signal. On the other hand, epitopes such as pS202 (CP13) or pS396 and pS404 (PHF-1) are abundant in the adult brain [28], and do not display overt non-specific signal in our experiments.

We were surprised to see a non-specific signal around 37 kD with the MC1 antibody in Tau KO mice (Fig. 3A). This antibody recognizes an abnormal conformation of tau encompassing amino acids 5–15 and 312–322 [29], [30]. We think that this non-specific signal is due to the knock-in of the EGFP coding sequence into the first exon disrupting the expression of the *Mapt* gene, and resulting in a chimeric protein with EGFP fused to the first 31 amino acids of tau [11]. Indeed, the same band was detected with an anti-GFP antibody and disappeared in the HS fraction (data not shown). These results indicate that, somehow, the fusion of EGFP to the small sequence of tau was able to mimic the MC1 epitope.

To help improve the detection of tau signal, we first used secondary antibodies designed to bind native Igs (TrueBlot). Because Igs coming from the samples are denatured, these antibodies recognize only the primary antibodies, and eliminate the interference of Igs heavy and light chains during the Western blot procedure [31]. Indeed, TrueBlot antibodies completely removed the non-specific signal from problematic primary antibodies in TKO, WT and 3xTg mice, allowing for the visualization of tau signal without interference. The use of TrueBlot antibodies did not necessitate an important modification of the standard Western blot procedure as they replaced conventional secondary antibodies. However, we observed that it was sometimes difficult to obtain a signal with TrueBlot secondary antibodies; hence, we had to increase the amount of protein being loaded or alter their incubation time from 1 h at room temperature for standard secondary antibodies to overnight or more at 4°C with TrueBlot.

As an alternative to TrueBlot, we also tested secondary antibodies designed to bind the light chain of Igs at 25 kDa and do not recognize the heavy chain at 50 kDa. These antibodies recognize only the primary antibodies and the light chain (LC) of Igs on the membrane, and eliminate the interference of Igs heavy chains in Western blot procedure. Indeed, anti-LC antibodies completely removed the non-specific signal from problematic primary antibodies in TKO, WT and 3xTg mice, allowing a visualization of tau signal without interference. Furthermore, the use of LC antibodies has several advantages versus TrueBlot antibodies: they are less expensive, can be diluted more, and incubated at room temperature for 1 h. However, these antibodies displayed a high non-specific band at 25 kDa that interfered with the automatic detection of tau signal during exposition with a quantitative imaging system, as these systems are often calibrated to stop the exposure process before the strongest signal on the membrane is outside the linear range of detection. We, therefore, recommend to mask the non-specific signal at 25 kDa, or to cut the membrane prior to incubation with secondary anti-LC antibodies, or to do incremental exposure to improve signal detection.

We also tested all of the monoclonal antibodies within a heat stable (HS) fraction. This method is routinely used by many groups for Western blotting [16], [32] or to measure total tau after passive immunization, as this procedure very effectively removes any mouse Igs (endogenous or exogenous) [13]. The heat-stable procedure is based on the fact that tau is an extremely heat stable protein [33]. Here, the non-specific signal seen in TKO mice was completely absent in the heat stable fraction, as expected. However, this technique also seems to decrease the overall tau signal and resolution on a Western blot, as evidenced by the observation that some antibodies failed to reveal all the bands of murine tau in the HS fraction. Furthermore, our results show that there is a significant fraction of tau lost into the pellet (Fig. 4A), and that this technique is not for studying proteins other than tau, such as kinases, as they were absent from the HS fraction (Fig. 4B, C).

We further tested two others techniques to clear Igs. We took advantages of the capacity of protein G and Dynabeads to bind the fragment crystallizable (F_C_) region of mouse Igs in order to clear the samples of endogenous Igs [15]. Results with cleared samples showed that the non-specific band in TKO mice was completely eliminated, further confirming that the non-specificity is due to mouse Igs. Both methods can be used to remove the non-specificity but they involve significant modification of Western blot procedures and are more time-consuming than the simple replacement of secondary antibodies. Furthermore, it is possible that some proteins of interest could form a complex with protein G or Dynabeads and be discarded in the pellet.

Finally, we tested some polyclonal antibodies and, as expected, our results indicate that these antibodies were not associated with specificity-related problems secondary to endogenous Igs. Nevertheless, in our study, some polyclonal antibodies, particularly the pS262 and pS409 antibodies, led to high non-specificity that obscured the tau signal detection. These antibodies displayed high non-specificity mainly due to their cross-reactivity with others proteins in the samples. While the HS treatment improved the phospho-tau signal of pS262, it did not for pS409. Indeed some studies have shown that pS409 is not phosphorylated in the human and rat adult brain, which could explain this problem with detection [27], [28].

In conclusion, this study provides evidence that both monoclonal and polyclonal antibodies can display non-specificity in mouse samples during Western blot analysis for tau protein. This is especially true for monoclonal antibodies directed at phosphoepitopes on tau and used in non-transgenic mice, particularly when the degree of phosphorylation of the epitopes is very low. In the present study, we described several easy, and inexpensive approaches that can be utilized to filter out the non-specific signal and improve tau signal specificity. Dynabeads, protein G, and the heat stable fraction all removed the non-specificity observed; however, we favor the use of secondary antibodies specific to native Igs or the use of anti-LC antibodies as these procedures interfered the least with our standard protocol and yielded good results. Finally, we stress the importance of using both negative (i.e., Tau KO) and positive controls (i.e., brains from hypothermic mice) to ascertain the specificity of a given signal during Western blot analysis.

## Supporting Information

Figure S1 **Quantifications of the Western blot displayed in** **Figure 2** **.** Results are expressed as percentage of WT group. Data are mean ± SD with n = 3 for each condition. Statistical analysis was performed with 1-way ANOVA followed by a Newman-Keuls Multiple Comparison Test. * denotes a significant difference compared to WT with P<0.05, ** with P<0.01 and *** with P<0.001. # denotes a significant difference compared to 3xTg-AD with P<0.05, ## with P<0.01 and ### with P<0.001.
(PDF)

Figure S2 **Quantifications of the Western blot displayed in** **Figure 3** **.** Results are expressed as percentage of WT group. Data are mean ± SD with n = 3 for each condition. Statistical analysis was performed with 1-way ANOVA followed by a Newman-Keuls Multiple Comparison Test. * denotes a significant difference compared to WT with P<0.05, ** with P<0.01 and *** with P<0.001. # denotes a significant difference compared to 3xTg-AD with P<0.05, ## with P<0.01 and ### with P<0.001. We were not able to quantify MC1 signal with TB antibodies and during HS fraction because of a poor ratio between MC1 and background signal.
(PDF)

Figure S3 **Quantifications of the Western blot displayed in** **Figure 5** **.** Results are expressed as percentage of WT group. Data are mean ± SD with n = 3 for each condition. Statistical analysis was performed with 1-way ANOVA followed by a Newman-Keuls Multiple Comparison Test. * denotes a significant difference compared to WT with P<0.05, ** with P<0.01 and *** with P<0.001. # denotes a significant difference compared to 3xTg-AD with P<0.05, ## with P<0.01 and ### with P<0.001.
(PDF)

Figure S4 **Quantifications of the Western blot displayed in** **Figure 6** **.** Results are expressed as percentage of WT group. Data are mean ± SD with n = 3 for each condition. Statistical analysis was performed with 1-way ANOVA followed by a Newman-Keuls Multiple Comparison Test. * denotes a significant difference compared to WT with P<0.05, ** with P<0.01 and *** with P<0.001. # denotes a significant difference compared to 3xTg-AD with P<0.05, ## with P<0.01 and ### with P<0.001.
(PDF)

Figure S5 **Quantifications of the Western blot displayed in** **Figure 7** **.** Results are expressed as percentage of WT group. Data are mean ± SD with n = 3 for each condition. Statistical analysis was performed with 1-way ANOVA followed by a Newman-Keuls Multiple Comparison Test. * denotes a significant difference compared to WT with P<0.05, ** with P<0.01 and *** with P<0.001. # denotes a significant difference compared to 3xTg-AD with P<0.05, ## with P<0.01 and ### with P<0.001.
(PDF)

## References

[1] Querfurth HW, LaFerla FM (2010) Alzheimer's disease. The New England journal of medicine 362: 329–344. 20107219 10.1056/NEJMra0909142

[2] Selkoe DJ (2001) Alzheimer's disease: genes, proteins, and therapy. Physiological reviews 81: 741–766. 11274343 10.1152/physrev.2001.81.2.741

[3] Grundke-Iqbal I, Iqbal K, Tung YC, Quinlan M, Wisniewski HM, et al (1986) Abnormal phosphorylation of the microtubule-associated protein tau (tau) in Alzheimer cytoskeletal pathology. Proceedings of the National Academy of Sciences of the United States of America 83: 4913–4917. 3088567 10.1073/pnas.83.13.4913 PMC323854

[4] Buée L, Bussière T, Buée-Scherrer V, Delacourte A, Hof PR (2000) Tau protein isoforms, phosphorylation and role in neurodegenerative disorders. Brain research Brain research reviews 33: 95–130. 10967355 10.1016/s0165-0173(00)00019-9

[5] Alonso AC, Zaidi T, Grundke-Iqbal I, Iqbal K (1994) Role of abnormally phosphorylated tau in the breakdown of microtubules in Alzheimer disease. Proceedings of the National Academy of Sciences of the United States of America 91: 5562–5566. 8202528 10.1073/pnas.91.12.5562 PMC44036

[6] Ballatore C, Lee VM-Y, Trojanowski JQ (2007) Tau-mediated neurodegeneration in Alzheimer's disease and related disorders. Nature reviews Neuroscience 8: 663–672. 17684513 10.1038/nrn2194

[7] St-Amour I, Pare I, Alata W, Coulombe K, Ringuette-Goulet C, et al.. (2013) Brain bioavailability of human intravenous immunoglobulin and its transport through the murine blood-brain barrier. Journal of cerebral blood flow and metabolism: official journal of the International Society of Cerebral Blood Flow and Metabolism. 10.1038/jcbfm.2013.160 PMC3851908 24045402

[8] Dawson HN, Ferreira A, Eyster MV, Ghoshal N, Binder LI, et al (2001) Inhibition of neuronal maturation in primary hippocampal neurons from tau deficient mice. Journal of cell science 114: 1179–1187. 11228161 10.1242/jcs.114.6.1179

[9] Oddo S, Caccamo A, Shepherd JD, Murphy MP, Golde TE, et al (2003) Triple-transgenic model of Alzheimer's disease with plaques and tangles: intracellular Abeta and synaptic dysfunction. Neuron 39: 409–421. 12895417 10.1016/s0896-6273(03)00434-3

[10] Planel E, Richter KEG, Nolan CE, Finley JE, Liu L, et al (2007) Anesthesia leads to tau hyperphosphorylation through inhibition of phosphatase activity by hypothermia. The Journal of neuroscience: the official journal of the Society for Neuroscience 27: 3090–3097. 17376970 10.1523/JNEUROSCI.4854-06.2007 PMC6672474

[11] Tucker KL, Meyer M, Barde YA (2001) Neurotrophins are required for nerve growth during development. Nature neuroscience 4: 29–37. 11135642 10.1038/82868

[12] Planel E, Yasutake K, Fujita SC, Ishiguro K (2001) Inhibition of protein phosphatase 2A overrides tau protein kinase I/glycogen synthase kinase 3 beta and cyclin-dependent kinase 5 inhibition and results in tau hyperphosphorylation in the hippocampus of starved mouse. The Journal of biological chemistry 276: 34298–34306. 11441005 10.1074/jbc.M102780200

[13] d'Abramo C, Acker CM, Jimenez HT, Davies P (2013) Tau passive immunotherapy in mutant P301L mice: antibody affinity versus specificity. PloS one 8: e62402. 23638068 10.1371/journal.pone.0062402 PMC3639259

[14] Jicha GA, Lane E, Vincent I, Otvos L Jr, Hoffmann R, et al (1997) A conformation- and phosphorylation-dependent antibody recognizing the paired helical filaments of Alzheimer's disease. Journal of neurochemistry 69: 2087–2095. 9349554 10.1046/j.1471-4159.1997.69052087.x

[15] Herskovits AZ, Davies P (2006) The regulation of tau phosphorylation by PCTAIRE 3: implications for the pathogenesis of Alzheimer's disease. Neurobiology of disease 23: 398–408. 16766195 10.1016/j.nbd.2006.04.004

[16] Duff K, Knight H, Refolo LM, Sanders S, Yu X, et al (2000) Characterization of pathology in transgenic mice over-expressing human genomic and cDNA tau transgenes. Neurobiology of disease 7: 87–98. 10783293 10.1006/nbdi.1999.0279

[17] Lewis J, Dickson DW, Lin WL, Chisholm L, Corral A, et al (2001) Enhanced neurofibrillary degeneration in transgenic mice expressing mutant tau and APP. Science 293: 1487–1491. 11520987 10.1126/science.1058189

[18] Neumann M, Sampathu DM, Kwong LK, Truax AC, Micsenyi MC, et al (2006) Ubiquitinated TDP-43 in frontotemporal lobar degeneration and amyotrophic lateral sclerosis. Science 314: 130–133. 17023659 10.1126/science.1134108

[19] Planel E, Krishnamurthy P, Miyasaka T, Liu L, Herman M, et al (2008) Anesthesia-induced hyperphosphorylation detaches 3-repeat tau from microtubules without affecting their stability in vivo. The Journal of neuroscience: the official journal of the Society for Neuroscience 28: 12798–12807. 19036972 10.1523/JNEUROSCI.4101-08.2008 PMC2610528

[20] Wittmann CW, Wszolek MF, Shulman JM, Salvaterra PM, Lewis J, et al (2001) Tauopathy in Drosophila: neurodegeneration without neurofibrillary tangles. Science 293: 711–714. 11408621 10.1126/science.1062382

[21] Zempel H, Thies E, Mandelkow E, Mandelkow EM (2010) Abeta oligomers cause localized Ca(2+) elevation, missorting of endogenous Tau into dendrites, Tau phosphorylation, and destruction of microtubules and spines. The Journal of neuroscience: the official journal of the Society for Neuroscience 30: 11938–11950. 20826658 10.1523/JNEUROSCI.2357-10.2010 PMC6633549

[22] Burnette WN (1981) “Western blotting”. : electrophoretic transfer of proteins from sodium dodecyl sulfate—polyacrylamide gels to unmodified nitrocellulose and radiographic detection with antibody and radioiodinated protein A. Analytical biochemistry 112: 195–203. 6266278 10.1016/0003-2697(81)90281-5

[23] Renart J, Reiser J, Stark GR (1979) Transfer of proteins from gels to diazobenzyloxymethyl-paper and detection with antisera: a method for studying antibody specificity and antigen structure. Proceedings of the National Academy of Sciences of the United States of America 76: 3116–3120. 91164 10.1073/pnas.76.7.3116 PMC383774

[24] Towbin H, Staehelin T, Gordon J (1979) Electrophoretic transfer of proteins from polyacrylamide gels to nitrocellulose sheets: procedure and some applications. Proceedings of the National Academy of Sciences of the United States of America 76: 4350–4354. 388439 10.1073/pnas.76.9.4350 PMC411572

[25] Porter RR (1959) The hydrolysis of rabbit y-globulin and antibodies with crystalline papain. The Biochemical journal 73: 119–126. 14434282 10.1042/bj0730119 PMC1197021

[26] Janke C, Beck M, Stahl T, Holzer M, Brauer K, et al (1999) Phylogenetic diversity of the expression of the microtubule-associated protein tau: implications for neurodegenerative disorders. Brain research Molecular brain research 68: 119–128. 10320789 10.1016/s0169-328x(99)00079-0

[27] Goedert M (1996) Tau protein and the neurofibrillary pathology of Alzheimer's disease. Annals of the New York Academy of Sciences 777: 121–131. 8624074 10.1111/j.1749-6632.1996.tb34410.x

[28] Yu Y, Run X, Liang Z, Li Y, Liu F, et al (2009) Developmental regulation of tau phosphorylation, tau kinases, and tau phosphatases. Journal of neurochemistry 108: 1480–1494. 19183272 10.1111/j.1471-4159.2009.05882.x PMC2676439

[29] Jicha GA, Bowser R, Kazam IG, Davies P (1997) Alz-50 and MC-1, a new monoclonal antibody raised to paired helical filaments, recognize conformational epitopes on recombinant tau. J Neurosci Res 48: 128–132. 9130141 10.1002/(sici)1097-4547(19970415)48:2<128::aid-jnr5>3.0.co;2-e

[30] Weaver CL, Espinoza M, Kress Y, Davies P (2000) Conformational change as one of the earliest alterations of tau in Alzheimer's disease. Neurobiol Aging 21: 719–727. 11016541 10.1016/s0197-4580(00)00157-3

[31] Zhang X, Ozawa Y, Lee H, Wen YD, Tan TH, et al (2005) Histone deacetylase 3 (HDAC3) activity is regulated by interaction with protein serine/threonine phosphatase 4. Genes & development 19: 827–839. 15805470 10.1101/gad.1286005 PMC1074320

[32] Couchie D, Nunez J (1985) Immunological characterization of microtubule-associated proteins specific for the immature brain. FEBS letters 188: 331–335. 2411600 10.1016/0014-5793(85)80397-5

[33] Weingarten MD, Lockwood AH, Hwo SY, Kirschner MW (1975) A protein factor essential for microtubule assembly. Proceedings of the National Academy of Sciences of the United States of America 72: 1858–1862. 1057175 10.1073/pnas.72.5.1858 PMC432646

