# Supplementary material for: Specificity of Anti-Tau Antibodies when Analyzing Mice Models of Alzheimer's Disease: Problems and Solutions
Source: PLoS One. 2014 May 2;9(5):e94251. doi: 10.1371/journal.pone.0094251 (PMC4008431; doi:10.1371/journal.pone.0094251)
Supplement: Figure S1 — Quantifications of the Western blot displayed in Figure 2 . Results are expressed as percentage of WT group. Data are mean ± SD with n = 3 for each condition. Statistical analysis was performed with 1-way ANOVA followed by a Newman-Keuls Multiple Comparison Test. * denotes a significant difference compared to WT with P<0.05, ** with P<0.01 and *** with P<0.001. # denotes a significant difference compared to 3xTg-AD with P<0.05, ## with P<0.01 and ### with P<0.001. (PDF) [file pone.0094251.s001.pdf]

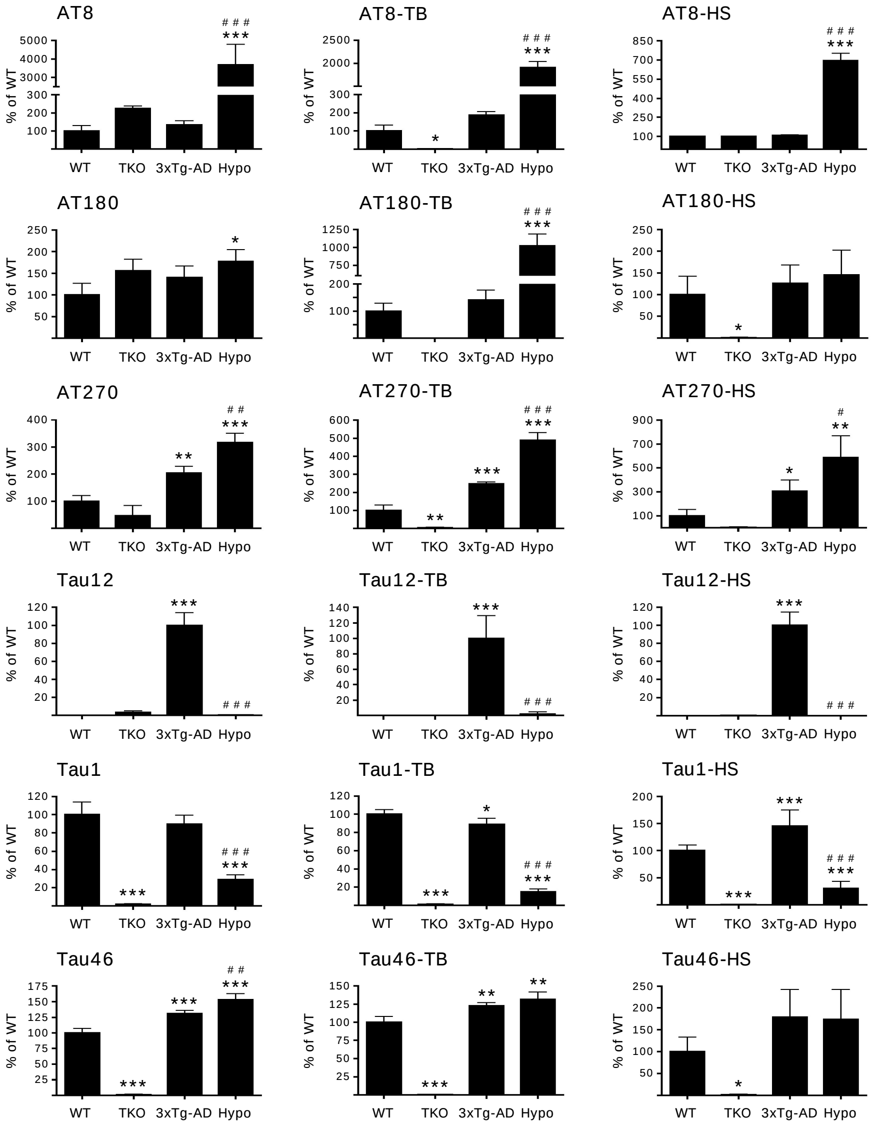

Figure S1. Quantifications of the Western blot displayed in Figure 2. Results are expressed as percentage of WT group. Data are mean ± SD with n = 3 for each condition. Statistical analysis was performed with 1-way ANOVA followed by a Newman-Keuls Multiple Comparison Test. * denotes a significant difference compared to WT with $P < 0.05$, ** with $P < 0.01$ and *** with $P < 0.001$. # denotes a significant difference compared to 3xTg-AD with $P < 0.05$, ## with $P < 0.01$ and ### with $P < 0.001$.
